# Differentiation of Benign from Malignant Adnexal Masses by Dynamic Contrast-Enhanced MRI (DCE-MRI): Quantitative and Semi-quantitative analysis at 3-Tesla MRI

**DOI:** 10.31557/APJCP.2019.20.4.1073

**Published:** 2019

**Authors:** Masoumeh Gity, Sara Parviz, Hamidreza Saligheh Rad, Anahita Fathi Kazerooni, Elham Shirali, Madjid Shakiba, Masoud Baikpour

**Affiliations:** 1 *Advanced Diagnostic and Interventional Radiology Research Center (ADIR),*; 2 *Department of Radiology, Medical Imaging Center,*; 3 *Quantitative MR Imaging and Spectroscopy Group, Research Center for Molecular and Cellular Imaging,*; 4 *Department of Medical Physics and Biomedical Engineering, School of Medicine,*; 5 *Department of Gynecology Oncology, Yas Hospital,*; 6 *School of Medicine Tehran University of Medical Sciences, Tehran, Iran. *

**Keywords:** Complex adnexal mass, dynamic contrast, enhanced MRI, pharmacokinetic modelling

## Abstract

**Background:**

To evaluate the utility of the pharmacokinetic modeling derived from dynamic contrast-enhanced magnetic resonance imaging (DCE-MRI) in differentiating benign from malignant adnexal masses.

**Methods:**

A total of 43 patients with 49 complex adnexal masses (27 benign, 3 borderline, and 19 malignant lesions) underwent preoperative DCE-MRI examinations on a 3 Tesla MRI. Using extended Tofts’ model, quantitative analysis was performed in the solid components of all tumors. Three pharmacokinetic parameters were defined as volume transfer coefficient (Ktrans), the rate constant (Kep), and the plasma volume (Vp). Semi-quantitative analysis was also performed and the values of relative signal intensity (SI rel) wash-in-rate (WIR), the initial area under the curve (iAUC60), time-to-peak (TTP) and wash-out-rate (WOR) were calculated. Receiver operating characteristic (ROC) curve analysis was performed to evaluate diagnostic characteristics of each DCE-MRI parameter in differentiating borderline/malignant tumors from benign lesions and to provide the optimal cutoff values for these variables.

**Results:**

SI rel had the highest diagnostic value (AUC=0.872; p<0.001; cut-off=121.4 associated with an overall accuracy=79.6%, sensitivity=95.5%, specificity=66.7%, NPV=94.8% and PPV=70.0%). Ktrans had the second highest AUC=0.836 (p<0.001; cut-off=0.034 associated with an overall accuracy=79.6%, sensitivity=86.4%, specificity=74.1%, NPV=87.0% and PPV=73.1%). The other factors found to be acceptable diagnostic parameters for borderline/malignant lesions included WIR (AUC=0.816; p<0.001), iAUC60 (AUC=0.808; p<0.001), Vp (AUC=0.795; p<0.001), SI max (AUC=0.737, p=0.005), SI peak (AUC=0.737; p=0.005) and Kep (AUC=0.681; p=0.031).

**Conclusion:**

Quantitative DCE-MRI is a relevant tool for differentiating benign from malignant adnexal masses. Among all the DCE parameters, SI rel and Ktrans are the most accurate discriminators.

## Introduction

Adnexal masses are among the leading indications of gynecological surgery (Mohaghegh and Rockall, 2012). As a result of the recent advances in surgical methods, different treatment approaches are available for adnexal lesions which are selected based on the probability of malignancy (Timmerman et al., 2005). In this regard, ultrasonography (US) has been commonly utilized as an initial imaging modality for estimating the risk of malignancy index (RMI) based on the specific features of malignancy on US (U-score), patient’s menopausal status and serum CA-125 levels (Jacobs et al., 1990; Arun-Muthuvel and Jaya, 2014; Ozbay et al., 2015). However, in some circumstances the lesion remains indeterminate and requires further assessments with other imaging modalities (Winarto et al., 2014; Malek et al., 2015).

Conventional contrast-enhanced magnetic resonance imaging (MRI) is indicated for all indeterminate adnexal masses, which provides an accuracy of 84-93% for differentiating benign and malignant lesions (Booth et al., 2008). However, its application is limited due to various reasons, particularly the dependence of its accuracy on the experience of reader (Hricak et al., 2000).

Recent studies have shown that a more accurate characterization of adnexal masses could be made using new objective MR parameters. For instance, dynamic contrast-enhanced MRI (DCE-MRI) is a technique that evaluates the leakage of contrast agent from capillaries into the extravascular extracellular space (Hricak et al., 2000) and its findings can be analyzed through three different approaches: descriptive, semi-quantitative and quantitative. Descriptive analysis is the most frequently used method while semi-quantitative and quantitative parameters are extracted using appropriate mathematical models (Thomassin-Naggara et al., 2008).

In the semi-quantitative analyses, changes of signal intensity is assessed over time and the individual variabilities such as cardiac output, arterial blood pressure and contrast agent dosage are not taken into account (Thomassin-Naggara et al., 2012; Tang et al., 2014). As previously established, among the semi-quantitative parameters, the maximal slope of the time-intensity curve is the most accurate for differentiating benign from malignant adnexal masses (Thomassin-Naggara et al., 2011). 

In order to address individual variabilities, a pharmacokinetic model is used, in which an arterial input function (AIF) is incorporated (Priest et al., 2010; Thomassin-Naggara et al., 2012). Although various studies have reported promising results for using the quantitative approach, but use of different methods has caused them to yield incongruent results (Priest et al., 2010; Thomassin-Naggara et al., 2012; Carter et al., 2013).

Currently there is not enough data available to determine which methodology and pharmacokinetic model is more accurate. Accordingly, this study was designed to evaluate the feasibility of semi-quantitative analysis along with the extended three-parametric two-compartment Tofts’ model, and to determine which parameter can differentiate between benign and malignant adnexal lesions more accurately.

## Materials and Methods

*Study Design and Sample Population*

Between January 2015 and March 2016, patients with a diagnosis of a complex adnexal mass on a trans-vaginal ultrasound, were enrolled in this prospective study. Complex solid cystic lesions with a solid component larger than 2 mm and multiloculated cysts with septae thicker than 2 mm were considered complex, and were included in the study. Patients with contraindications to contrast administration based on their recent serum creatinine levels were excluded. All participants underwent preoperative MRI and DCE-MRI, and were scheduled for surgical resection within 40 days of imaging. 

Eventually, a total of 47 patients were recruited, of which 4 were excluded from the study; three due to consideration of non-surgical treatment and one due to poor quality imaging. Of the remaining 43 patients, 12 women had cystectomy, 20 women had unilateral salpingo-oophorectomy and 10 subjects had bilateral salpingo-oophorectomy with hysterectomy and omentectomy. For one patient, resection of the tumor was not possible because the tumor was highly vascular and fragile; therefore, only open biopsy was performed and histopathological assessment of the lesion was consistent with ovarian Primitive neuroectodermal tumor (PNET).

Six patients had bilateral lesions including two papillary serous carcinomas, one ovarian lymphoma, one ovarian metastasis, one mucinous cyst adenocarcinoma, and one ovarian PNET. In bilateral tumors, each lesion was considered as one individual study case. Thus, a total of 43 patients with 49 adnexal masses were included in our analyses. Table 1 presents the histopathological findings of the ovarian tumors.

*Imaging technique*

All MRI examinations were performed on a 3 Tesla MR unit (Magnetom Avanto; Siemens, Erlangen, Germany) with a phased-array pelvic coil. The range of scan was between the umbilicus and the pubic symphysis. The patients were fasting for at least 6 hours before the imaging. An antispasmodic agent (1ml of Hyoscine) was given by intramuscular injection immediately before image acquisition to minimize the artifacts caused by bowel peristalsis. An abdominal belt was also used to help reduce respiratory motion-related artifacts. 

The sequences acquired before the injection were as follows: Sagittal T2-weighted fast spin-echo (TR=5,050, TE=121, slice thickness=5 mm, gap=1 mm, FOV=280x225, matrix=512x245), axial T2-weighted fast spin-echo (TR=6,790, TE=89, slice thickness=5 mm, gap=1 mm, FOV=370x275, matrix=512x187) and axial gradient-echo T1-weighted sequences with and without fat suppression (TR=170, TE=4.76, flip angle=70°, slice thickness=5 mm, gap=1 mm, FOV=320x280, matrix=246x145).

**Table 1:** Definition of the Semi-Quantitative Parameters

Parameter	Definition
SI_max_	Maximum signal intensity over the time course of the enhancement curve
SI_rel_	Relative signal intensity = (SI_max_-SI_0_)/SI_0_×100
TTP	Time-to-peak
WIR	Wash-in-rate = (SI_max_-SI_0_)/TTP
WOR	Wash-out-rate = (SI_max_-SI_end_)/(SI_max_-SI_0_)
iAUC_60_	Initial area under the curve in the initial 60 seconds.

**Table 2:** Distribution of Histopathological Diagnoses Based on the WHO Classification

Malignancy	Diagnosis	Frequency (Percent)
Benign (n=27)	Broad ligament fibroma	2 (4.1%)
	Dermoid cyst	2 (4.1%)
	Endometrioma	16 (32.6%)
	Hydrosalpynx	2 (4.1%)
	Mucinous cystadenoma	2 (4.1%)
	Serous cystadenofibroma	1 (2.0%)
	Serous cystadenoma	2 (4.1%)
Borderline (n=3)	Borderline mucinous cystadenoma	2 (4.1%)
	Borderline papillary serous cystadenoma	1 (2.0%)
Malignant (n=19)	Dysgerminoma	2 (4.1%)
	Endodermal sinus tumor	1 (2.0%)
	Endometrioid adenocarcinoma	2 (4.1%)
	Krukenburg tumor	2 (4.1%)
	Ovarian Lymphoma	2 (4.1%)
	Mucinous cystadenocarcinoma	2 (4.1%)
	Papillary serous cystadenocarcinoma	5 (10.2%)
	PNET	2 (4.1%)
	Serous cystadenocarcinoma	1 (2.0%)
Total		49 (100.0%)

**Figure:**
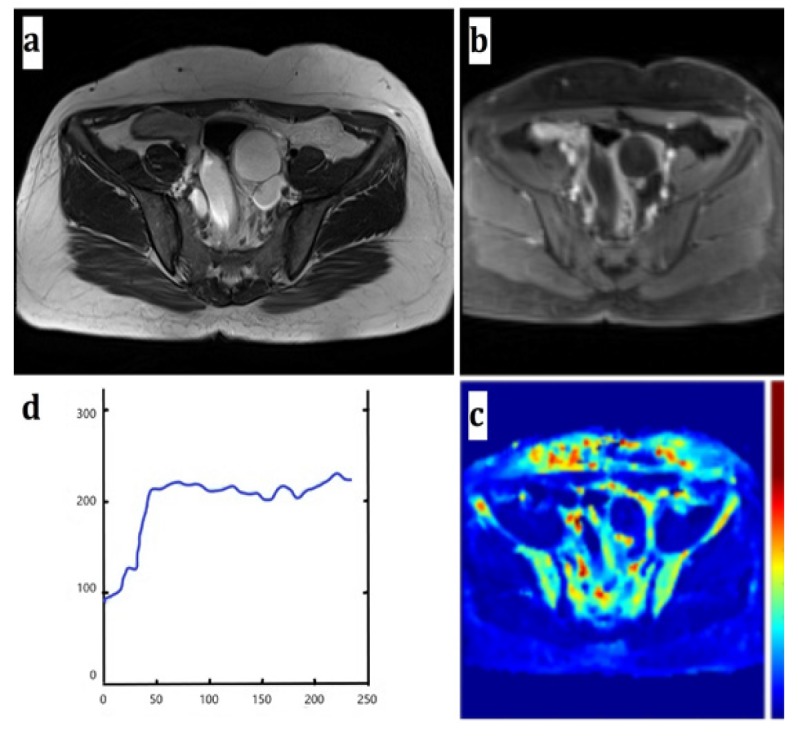
Figure 1Region of Interest (ROI) drawn on a benign adnexal mass (a serous cystadenoma) (a) Axial T2-weighted image (b) Axial DCE T1-weighted image (c) Ktrans coded color map, ROI was drawn on a small enhancing papillary projection of the tumor. (d) Benign enhancement curve and pharmacokinetic and semi-quantitative parameters

**Figure 2:**
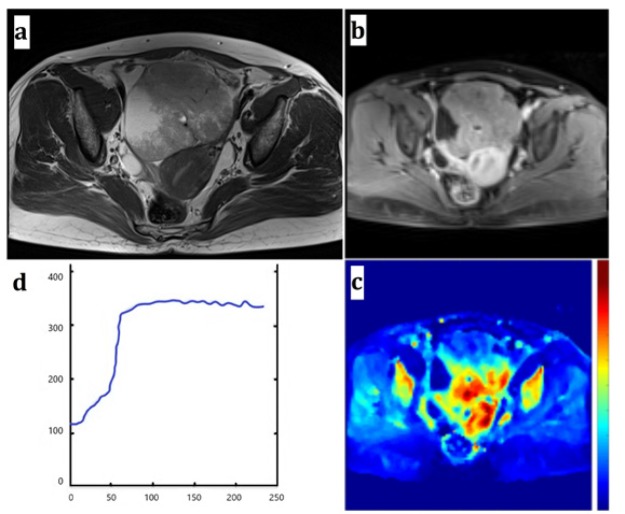
Region of Interest (ROI) drawn on a malignant adnexal mass (an endometrioid carcinoma) (a) Axial T2-weighted image (b) Axial DCE T1-weighted image (c) Ktrans coded color map, ROI was drawn on a solid portion of the tumor. (d) Malignant enhancement curve and pharmacokinetic and semi-quantitative parameters

**Figure 3:**
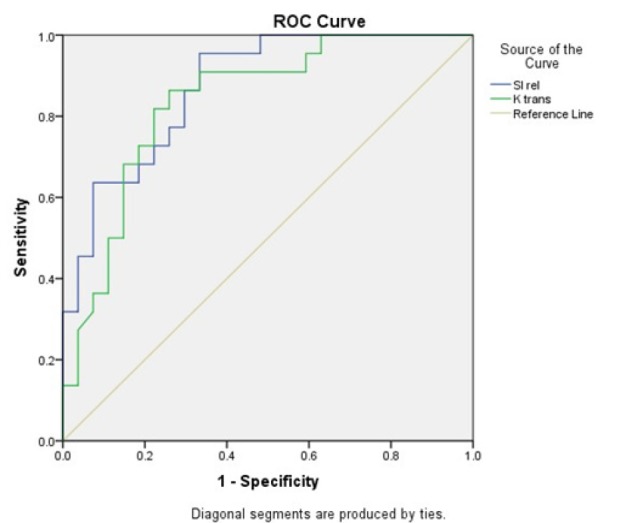
ROC Curve for SI rel and Ktrans

**Table 3:** Comparison between the Mean Values of the PK and Semi-Quantitative Parameters between Different Groups

Parameter	Pathology			P value^a^	P value^b^	P value^c^
	Mean (Standard Deviation)					
	Benign (n=27)	Borderline (n=3)	Malignant (n=19)			
Age	39.93 (8.38)	44.67 (16.29)	39.05 (12.51)	0.697	0.778	0.972
Ktrans	0.03 (0.02)	0.03 (0.01)	0.05 (0.01)	<0.001	<0.001	<0.001
Kep	1.89 (3.16)	3.67 (2.58)	5.38 (11.81)	0.333	0.149	0.148
Ve	0.03 (0.03)	0.02 (0.02)	0.04 (0.03)	0.59	0.659	0.873
Vp	0.10 (0.07)	0.25 (0.07)	0.22 (0.14)	<0.001	<0.001	<0.001
TTP	98.24 (39.09)	134.27 (101.21)	100.00 (45.69)	0.441	0.892	0.642
SI max	242.14 (112.02)	321.06 (120.34)	331.20 (90.51)	0.02	0.005	0.004
SI peak	217.93 (100.81)	288.95 (108.31)	298.08 (81.46)	0.02	0.005	0.004
SI rel	97.36 (50.69)	173.53 (36.29)	194.92 (79.29)	<0.001	<0.001	<0.001
WIR	0.66 (0.55)	1.07 (0.58)	1.61 (1.41)	0.009	0.003	0.007
WOR	0.13 (0.22)	0.12 (0.05)	0.07 (0.08)	0.516	0.207	0.258
iAUC60	1,751.26 (1543.70)	4,281.02 (2127.19)	3,944.73 (2356.96)	0.001	0.001	<0.001

^a^, Comparison between the three groups of benign, borderline and malignant; ^b^, Comparison between the two groups of benign and malignant; ^c^, Comparison between the two groups of benign and borderline/malignant.

**Table 4:** Diagnostic Characteristics of Evaluated Parameters for Differentiating Malignant/Borderline Tumors from benign Lesions

Variable	AUC	P-value	Cut off	Accuracy	Sensitivity	Specificity	NPV	PPV
Ktrans	0.836	<0.001	0.034	79.6	86.4	74.1	87	73.1
Kep	0.681	0.031	1.07	65.3	72.7	59.3	72.7	59.3
Ve	0.55	0.553	-	-	-	-	-	-
Vp	0.795	<0.001	0.145	79.6	72.7	85.2	79.3	80
TTP	0.501	0.992	-	-	-	-	-	-
SI max	0.737	0.005	259.1	69.4	77.3	63	77.3	63
SI peak	0.737	0.005	233.2	69.4	77.3	63	77.3	63
SI rel	0.872	<0.001	121.4	79.6	95.5	66.7	94.8	70
WIR	0.816	<0.001	0.588	69.4	86.4	55.6	83.4	61.3
WOR	0.489	0.896	-	-	-	-	-	-
iAUC_60_	0.808	<0.001	1894	77.5	86.4	70.4	86.4	70.4

Then, a dose of 0.1 mmol.kg−1 of gadoterate meglumine (Dotarem, Guerbet, USA) was administered with an MR-compatible power injector at a rate of 2 mL.s−1, followed by a bolus of 20 mL saline solution (0.9%).

DCE sequences were obtained from the tumor’s solid components (papillary projection, solid nodule or thickened septa) based on the findings of initial non-enhanced sequences, and consisted of axial DCE T1-weighted gradient-echo sequence (3D Turbo FLASH) (TR=3.16, TE=1.03, flip angle=8°, slice thickness=4 mm, gap=1 mm, FO=320x320, matrix=128x128). DCE images were acquired at 7.6/frame intervals, beginning 10 seconds before the bolus injection for a total of 380s (50 time frames). Finally, delayed enhanced axial and sagittal T1-weighted gradient-echo images with breath-hold were obtained 6 min after injection.

*Imaging analysis*

All images were reviewed by two experienced radiologists blinded to the results of histopathological assessments (M.G with 10 years and S.P with 4 years of experience in gynecologic imaging). Color-coded Ktrans maps were created for all slices of adnexal masses, and regions of interest (ROIs) were manually placed on the hot-spots of the tumor. Three small ROIs were placed on the most enhancing part of the lesion, trying to include only a few pixels so that it does not involve the adjacent tissues.

*Pharmacokinetic Analysis*

Pharmacokinetic (PK) image analysis was performed, assuming a low-dose protocol and subsequently, presence of a linear relationship between the changes of signal intensity and concentration of the contrast agent within the ROI. Quantitative DCE parameters including Ktrans (the volume transfer rate), Kep (the rate constant) and vp (the fraction of plasma volume) were computed for the selected ROIs based on extended Tofts PK model (Tofts et al., 1999):


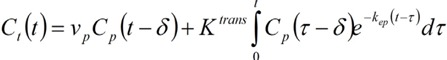


In the above equation, δ is the time lag between the onset of the concentration rise in the tissue and the beginning of arterial input concentration, Cp represents the concentration in the plasma which is calculated assuming a hematocrit level (HCT) of 45% and using (1-HCT) Cb. AIF or Cb was obtained through a population-averaged model proposed by Weinmann. To acquire the best fit, the population-based AIF was calibrated to the AIF obtained from 10 randomly selected patients among our study population, in whom the iliac artery was selected as the region of arterial input. The time lag, δ, was selected automatically by increasing the parameter by 1 unit in a range of values repeatedly and was selected as the value returning the best results for goodness of fit evaluation parameters. All the mentioned quantitative analyses were performed by in-house MATLAB scripts (Figures 1 and 2). 

*Semi-Quantitative Analysis*

Several DCE-MRI curve descriptors including, maximum signal intensity (SI max), relative signal enhancement (SI rel), time to peak (TTP), wash-in-rate (WIR), wash-out-rate (WOR), and initial area under the time-intensity curve (iAUC60) were calculated as semi-quantitative parameters (Table 1).

*Statistical analysis*

Mean values of individual parameters were compared among benign, borderline and malignant adnexal lesions using One-way ANOVA test. The significance of differences between benign and malignant lesions, and benign and borderline/malignant tumors were also assessed using independent two-tailed Student’s t-test for normally distributed data or otherwise by Mann-Whitney U-test, after examining the normality of data for variables based on Shapiro-Wilk test. A p value of less than 0.05 was considered statistically significant. Receiver operating characteristic (ROC) curve analysis was also performed to evaluate diagnostic characteristics of each DCE-MRI parameter in differentiating borderline/malignant tumors from benign lesions and, to provide the optimal cutoff values for these variables. All statistical calculations were performed using SPSS 23.0 for Windows platform (SPSS, Chicago, IL, USA, 2015). 

*Ethical consideration*

Our institutional review board of the university hospital approved the study protocol. The objectives and methods of the survey were thoroughly explained for the subjects invited for participation. They were reassured that their inclusion in the study will not affect their treatment, will not pose additional charges to them and their information will be considered confidential and used anonymously, and only the main researchers will have access to them. Eventually, an informed written consent was obtained from all patients willing to participate. The study protocol was in accordance to the guidelines of the Helsinki’s Declaration. 

## Results

Data from a total of 43 patients with 49 adnexal masses were included in our analyses, which comprised of 27 benign (55.1%), 3 borderline (6.1%) and 19 malignant (38.8%) lesions. Considering the invasive potential of borderline tumors, we categorized the three borderline cases in the same group as malignant lesions for analysis purposes. The mean age of the patients was 39.9±10.5 years, ranging from 18 to 59 years. Table 2 presents the distribution of histopathological diagnoses in the sample population.

Table 3 shows the values of age and other evaluated parameters in the three groups of benign, borderline and malignant lesions, and assesses the significance of differences between these groups. The first column of the p values refers to the comparison between the three groups via ANOVA test, the second column presents the p values for the comparison between benign and malignant groups and the third column refers to the comparison between benign and borderline/malignant categories. As can be seen, there were no significant differences between these groups regarding age, Kep, Ve, TTP and WOR. On the other hand, Ktrans, Vp, SI max, SI peak, SI rel, WIR and iAUC60 were all found to be significantly higher in the malignant lesions compared to benign tumors. Inclusion of the borderline lesions in the malignant group also yielded similar results. 

Diagnostic characteristics of evaluated parameters for differentiating borderline/malignant lesions from benign tumors are presented in Table 4. The highest AUC was calculated for the SI rel (AUC=0.872; p<0.001) that provides an overall accuracy=79.6%, sensitivity=95.5%, specificity=66.7%, NPV=94.8% and PPV=70.0%, with a cut-off value of 121.4. Ktrans had the second highest AUC of 0.836 (p<0.001), which considering a cut-off value of 0.034 translates to an accuracy=79.6%, sensitivity=86.4%, specificity=74.1%, NPV=87.0% and PPV=73.1% (Figure 3). The other factors found to be acceptable diagnostic parameters for borderline/malignant lesions included WIR (AUC=0.816; p<0.001), iAUC60 (AUC=0.808; p<0.001), Vp (AUC=0.795; p<0.001), SI max (AUC=0.737, p=0.005), SI peak (AUC=0.737; p=0.005) and Kep (AUC=0.681; p=0.031). 

## Discussion

Our study demonstrates the feasibility of performing PK analysis using the extended three-parametric two-compartment Tofts’ model for differentiating between malignant and benign adnexal masses. Previous studies have also shown the usefulness of PK parameters in evaluating adnexal masses. The fact that PK analysis can be used in this setting owes to the presence of disorganized neoangiogenetic vessels in malignant tumors that lack muscular coating, which allows for the contrast leakage into extravascular extracellular space (Abu-Jawdeh et al., 1996).

In one of the previous studies on the same topic, using a four-parametric two-compartment model on a 1.5 Tesla MRI machine, Thomassin-Naggara et al. found a higher value of the tissue blood flow (FT), a higher blood fraction volume (Vb), a lower interstitial volume (Ve) and a higher relative AUC (rAUC) in malignant adnexal tumors compared to benign lesions (Thomassin-Naggara et al., 2012). Their results also showed that the borderline ovarian tumors have higher FT and lower lag time (Dt) in comparison with the malignant lesions.

Carter et al., (2013) evaluated the PK parameters using extended Tofts’ model on a 3 Tesla MRI scanner. Having placed the ROI on the most solid area of the adnexal masses, the results of their study showed that most DCE parameters were significantly higher in malignant tumors, among which Kep was the most accurate. The present survey was also based on the three-parametric two-compartmental extended Tofts’ model in a 3 Tesla MRI machine. Using a high magnetic field in these two studies provided the advantage of increased signal to noise ratio (Priest et al., 2010). Our study was consistent with previous reports in demonstrating the feasibility of PK parameters in differentiating malignant from benign adnexal masses; however, incompatible with the findings of Carter et al., (2013) our results revealed that among the PK parameters the most accurate one was the Ktrans, found to be higher in malignant lesions. 

In this study we also evaluated the semi-quantitative DCE-MRI parameters in these adnexal lesions. As shown previously by Bernardin et al., (2012) among the semi-quantitative parameters the malignant tumors had higher values of SI max, SI rel and WIR, and the latter was the most accurate predictive parameter. We also found SI max, SI peak, SI rel and WIR to be significantly higher in malignant lesions compared to benign tumors, and we included iAUC60 in our analyses as well, which was similarly higher in malignant masses. Incompatible with their’s results, we showed that the most accurate parameter among these variables for predicting malignant lesions is the SI rel, which was also found to be a better predictive factor than the PK parameters we assessed with an AUC of 0.872. The overall accuracy of SI rel was calculated to be 79.6% with a sensitivity of 95.5% and a specificity of 66.7%, assuming a cut-off value of 121.4. 

In our study population there were three borderline tumors, two of which had Ktrans values in the malignant range (histopathological reports of both showed mucinous type tumor), and one had a low Ktrans value in the range of benign lesions with a histopathological report of papillary serous type tumor. There was also an ovarian PNET in our study population, which is a very rare pelvic tumor of young women treated initially by non-surgical interventions (Yousefi et al., 2014). PK analyses of this tumor showed a Ktrans value of 0.264, which was much higher than other malignant tumors in our study population. This finding can be attributed to the highly fragile vessels formed in this type of malignancy.

We included 2 dermoid cysts and 16 cases with endometriomas. Although these lesions generally have no solid components, have thin enhancing walls and are easily diagnosed on conventional MRI images, all the included cases in the current study met the aforementioned inclusion criteria and were eligible for inclusion. 

The small sample size in the present study was one of the limitations of this survey and so further investigations with larger sample populations should be conducted to confirm or negate our findings. Moreover, in this study we classified borderline tumors in the same group as the malignant lesions. Although these two categories may share some characteristics, but could be distinguished from each other using PK parameters. Therefore, it is suggested that further studies include more patients with borderline tumors so that a more comprehensive comparison could be made between their characteristics and that of malignant lesions. 

In conclusion, this study demonstrated that PK modeling of DCE-MRI is a relevant tool for estimating the likelihood of malignancy in the adnexal tumors. Our results also showed that SI rel and Ktrans have higher diagnostic values over other quantitative PK and semi-quantitative parameters in differentiating benign and malignant adnexal tumors.
